# Estimating Cancer Penetrance in Carriers of *BRCA2* Pathogenic Variants Using Cancer‐Specific Polygenic Scores

**DOI:** 10.1002/cam4.70990

**Published:** 2025-06-03

**Authors:** Brendan Prassas, Zhuqing Shi, Huy Tran, Jun Wei, Chi‐Hsiung Wang, Andrew S. Rifkin, Annabelle Ashworth, S. Lilly Zheng, Ashley J. Mulford, Alan R. Sanders, Catherine E. Pesce, Brian T. Helfand, Henry M. Dunnenberger, David Duggan, Peter J. Hulick, Allison DePersia, Jianfeng Xu

**Affiliations:** ^1^ Program for Genomic Translational Research NorthShore University HealthSystem (Endeavor Health) Evanston Illinois USA; ^2^ Genomic Health Initiative NorthShore University HealthSystem (Endeavor Health) Evanston Illinois USA; ^3^ University of Chicago Pritzker School of Medicine Chicago Illinois USA; ^4^ Department of Surgery NorthShore University HealthSystem (Endeavor Health) Evanston Illinois USA; ^5^ Neaman Center for Personalized Medicine NorthShore University HealthSystem (Endeavor Health) Evanston Illinois USA; ^6^ Translational Genomics Research Institute, Part of City of Hope Phoenix Arizona USA

**Keywords:** *BRCA2*, breast cancer (female and male), hereditary breast and ovarian cancer syndrome (HBOC), liver cancer, lung cancer, oral cancer, ovarian cancer, pancreatic cancer, polygenic score, prostate cancer

## Abstract

**Introduction:**

*BRCA2* is a causal gene for hereditary breast and ovarian cancer (HBOC) syndrome. However, its association with other cancers and interplay with polygenic scores (PGS) remains unclear.

**Methods:**

An observational cohort study for the diagnosis of various cancers in the UK Biobank (UKB, *N* = 453,541) were recruited at ages of 40–69 years Association of germline pathogenic variants (PVs) in *BRCA2* and published cancer‐specific PGS with cancer risk was tested using Cox proportional hazards model.

**Results:**

The median age and interquartile range (IQR) of participants at the analysis was 58.34 (50.60–63.74) years. Carriers of *BRCA2* PVs (*N* = 1629) had a significantly increased risk for four core HBOC‐associated cancers (breast, ovarian, pancreatic, and prostate) and six additional types of cancer (lung, oral, small intestine, larynx, liver, and mesothelioma), hazard ratio (HR) > 2.37, all *p*s < 0.001. For eight cancers where cancer‐specific PGS is available, each PGS was significantly associated with its respective cancer risk and independent of *BRCA2*, HR > 1.25 for 1 unit increase in standard deviation, all *p*s < 0.001. For female breast and prostate cancer, a significant interaction between *BRCA2* and PGS was found (HR < 0.83, *p* < 0.05); the effect of PGS on cancer risk was weaker in carriers than noncarriers. The probability of cancer by age 75 years (P_75_) for these 10 cancers increased with higher PGS deciles in both carriers and noncarriers. For several cancers, the P_75_ in carriers with the lowest PGS decile was lower than that of noncarriers with the highest PGS decile.

**Conclusions:**

*BRCA2* PVs increase risk beyond core HBOC cancers and their risks are modified by cancer‐specific PGS. These results suggest that genetic counseling of *BRCA2* PV carriers may extend to cancers beyond core HBOC syndrome and incorporate cancer‐specific PGS in estimating their penetrance.

## 1 Introduction

*BRCA2* is a gene that encodes a protein involved in DNA homologous recombination repair and plays a crucial role in maintaining genomic stability [1, 2]. Pathogenic variants (PVs) in *BRCA2* are a major cause of hereditary breast and ovarian cancer (HBOC) syndrome, a condition characterized by a significantly increased risk of developing breast, ovarian, pancreatic, and prostate cancers [3]. *BRCA2* PVs may also increase the risk for other cancers, including uterine cancer, leukemia, melanoma, and esophagus cancer, though evidence remains inconclusive [4, 5, 6, 7, 8, 9, 10, 11, 12].

In addition to the uncertainty about the spectrum of cancers associated with *BRCA2*, the reported penetrance for each cancer type in carriers varies considerably, ranging from 30% to 80% for breast cancer, 13%–29% for ovarian cancer, 5%–10% for pancreatic cancer, and 19%–61% for prostate cancer [13, 14, 15, 16, 17]. A major factor for the wide ranges of penetrance estimates is the relatively low frequency of *BRCA2* PV carriers, estimated at 1:400 to 1:500 in the general population [18]. Another major source of variability in risk estimates is from various study designs where those based on highly selected subjects with a strong family history of HBOC and early onset of cancer often reported higher risks than population based studies, due to selection bias [19]. Furthermore, other nongenetic risk factors such as diet, physical activity, alcohol and tobacco, hormonal exposures, as well as radiation and pollution may also contribute to the variability of cancer risk among *BRCA2* PV carriers [20].

Furthermore, overwhelming evidence in the last two decades suggests that the penetrance of cancer is also influenced by a large number of common cancer‐specific risk‐associated genetic variants in the genome [21]. Although their individual effects on cancer risk are modest, they have a stronger cumulative effect on cancer risk, which can be measured using a polygenic score (PGS). Cancer‐specific PGSs have been developed and validated for predicting genetic susceptibility for various cancers [22, 23, 24, 25, 26, 27]. Importantly, several studies also suggested that PGS can modify the penetrance of *BRCA2* PV carriers for several cancers, including breast and prostate cancer [28, 29, 30, 31, 32, 33, 34, 35, 36, 37].

The objectives of this study were to systematically assess the spectrum of *BRCA2*‐associated cancers and estimate the penetrance of *BRCA2* PVs for these cancers in a large population based cohort, both independently and jointly with PGS.

## 2 Methods

### 2.1 Participants

Study subjects were participants from the UK Biobank (UKB). The UKB is a large population based cohort of participants aged between 40 and 69 years old at recruitment from across the United Kingdom [38]. Cancer diagnoses, based on International Classification of Diseases‐10 (ICD‐10) codes (C00–C96), were obtained from self‐report, inpatient diagnosis, and the UK cancer registry (the last date of access was June 12, 2023). Genetic data from whole exome sequencing (WES) and SNP array (as well as imputed SNPs) were accessible. Access to the UKB data was approved under the application number 50295.

Genetic probabilities for Finnish (FIN), non‐Finnish European (NFE), Ashkenazi Jewish (ASJ), East Asian (EAS), African (AFR), admixed American (AMR), and South Asian (SAS) were calculated for each subject based on the top 20 principal components (PCs) of 16,109 ancestry informative markers across the genome.

### 2.2 Genetic Risk Factors

All candidate protein‐altered variants in exons, exon–intron junctions, and untranslated regions (UTRs) of *BRCA2* were identified from WES data. Their pathogenicity was annotated based on the ClinGen ENIGMA *BRCA1* and *BRCA2* Variant Curation Expert Panel (August 2023) [39]. Identified pathogenic and likely pathogenic (P/LP) variants in the UKB are listed in Table S1 and are grouped as PVs. Cancer‐specific pan‐ancestry polygenic scores (PGSs) were selected based on published methods in the PGS Catalog (Table S2) [22, 23, 24, 25, 26, 27, 40]. When multiple PGS methods were available for a specific type of cancer, the PGS with the largest number of subjects of multiancestry background was selected. The raw PGS score of each subject was first calculated based on their weighted genotypes. Ancestry‐adjusted PGS was then calculated using the first four PCs, as described previously [41].

### 2.3 Statistical Methods

We followed the method described by Fahed et al. to test the association of cancer risk with *BRCA2* PVs and PGS [31]. Briefly, a Cox proportional hazards model was used for the time to event (each specific type of cancer), where the timescale was the age at which the diagnosis was first ascertained in cases and the age at the most recent follow‐up in controls. These models included carrier status, sex (except for gender‐limited cancers, which were restricted to females and males, respectively), and the first 10 principal components of ancestry as covariates. Hazard ratio (HR) and 95% confidence interval (CI) were estimated for each type of cancer, and those with *p* < 0.001 (Bonferroni correction for testing 53 types of cancer, 0.05/53) were considered significant. For these *BRCA2*‐associated cancers, cancer‐specific PGS (if available) were added to the model to test the independent and interaction effect with *BRCA2* PVs (carrier status × PGS). The probability of developing cancer by a given age (e.g., P_75_ for age 75) was estimated as a function of PV carrier status and PGS, with all other covariates standardized to their mean values in the model. The probability of disease by time *t* was estimated by *F*(*t*) = 1 − *S*(*t*), where *S*(*t*) is the survivor function, estimated from the R survival package (v4.3.3; R Core Team 2024 for R).

## 3 Results

### 3.1 Characteristics of Subjects

All participants in the UKB with genetic data (*BRCA2* and PGS) were included in the analysis, including 453,541 subjects (Table S3). The median age and interquartile range (IQR) at recruitment was 58.34 (50.60–63.74) years. The vast majority of subjects in the cohort were of European ancestry (93.93%) and 54.23% were female.

### 3.2 Carrier Rate of BRCA2 PVs

Among the 453,541 subjects, 1629 (0.36%) carriers of *BRCA2* PVs were identified, all heterozygous. The carrier rate was disproportionately high in the ASJ ancestry population (1.24%), and lower in other ancestry populations, including 0.43% for EAS, 0.36% for both NFE and AFR, 0.26% for SAS, and 0.13% for AMR (Table S3).

### 3.3 BRCA2 PVs and the Spectrum of Cancer Risk

Carriers of *BRCA2* PVs had a significantly higher prevalence of any cancer (41.80%) compared to noncarriers (22.39%), HR (95% CI) was 2.29 (2.12–2.47), *p* < 0.001. Notably, carriers were more likely to be diagnosed with two or more types of cancer (21.36%) than noncarriers (7.39%), with an HR of 3.53 (3.18–3.92), *p* < 0.001 (Tables 1 and S4).

**TABLE 1:** Association of *BRCA2* PVs with cancer risk in the UKB.

	Cancer in noncarriers, *N* (%)	Cancer in carriers, *N* (%)	HR (95% CI) ^a^, ^b^	*p* value ^a^, ^b^
Any cancers	101,162/451,912 (22.39)	681/1629 (41.8)	2.29 (2.12–2.47)	8.76E‐103
Single site	68,079/101,843 (66.85)	333/1629 (20.44)	1.67 (1.50–1.86)	6.96E‐21
Multiple sites	33,764/101,843 (33.15)	348/1629 (21.36)	3.53 (3.18–3.92)	4.18E‐121
By cancer site				
Core HBOC cancers				
Breast (female), C50 ^c^	17,553/245,099 (7.16)	252/863 (29.2)	4.89 (4.32–5.54)	4.84E‐138
Breast (male), C50 ^d^	130/206,813 (0.06)	7/766 (0.91)	15.42 (7.20–33.01)	1.90E‐12
Ovary, C56	1963/245,099 (0.8)	69/863 (8)	10.89 (8.56–13.85)	1.78E‐84
Pancreas, C25	1711/451,912 (0.38)	20/1629 (1.23)	3.57 (2.30–5.55)	1.51E‐08
Prostate, C61	14,809/206,813 (7.16)	115/766 (15.01)	2.37 (1.98–2.85)	2.66E‐20
Beyond core HBOC cancers				
Larynx, C32, C33	456/451,912 (0.1)	6/1629 (0.37)	3.89 (1.74–8.70)	9.55E‐04
Liver, C22	1047/451,912 (0.23)	11/1629 (0.68)	3.17 (1.75–5.74)	1.41E‐04
Lung, C34	5302/451,912 (1.17)	46/1629 (2.82)	2.69 (2.01–3.59)	2.48E‐11
Mesothelioma, C45–C49	1580/451,912 (0.35)	22/1629 (1.35)	4.17 (2.73–6.35)	3.04E‐11
Oral, C00–C14	1856/451,912 (0.41)	18/1629 (1.1)	2.80 (1.76–4.46)	1.35E‐05
Small intestine, C17	462/451,912 (0.1)	6/1629 (0.37)	3.90 (1.74–8.72)	9.33E‐04
Ill‐defined, secondary, unspecified sites, C76–C80	20,887/451,912 (4.62)	290/1629 (17.8)	4.40 (3.92–4.94)	1.57E‐138

^a^ Adjusted for date of birth, gender (except for gender‐specific cancers) and genetic background (top 4 PCs).

^b^ Time from birth date to event/death/the end of follow‐up (June 12, 2023).

^c^ Tested in female subjects.

^d^ Tested in male subjects.

For each individual cancer type, carriers of *BRCA2* PVs were significantly associated with increased risk for each of the four core HBOC cancers. The HR (95% CI) was 4.89 (4.32–5.54) for female breast, 15.42 (7.20–33.01) for male breast, 10.89 (8.56–13.85) for ovarian cancer, 3.57 (2.30–5.55) for pancreatic cancer, and 2.37 (1.98–2.85) for prostate cancer, all *p* < 0.001. Additionally, carriers were significantly associated with increased risks for six other cancer types, including cancer of larynx (HR = 3.89), liver (HR = 3.17), lung (HR = 2.69), mesothelioma (HR = 4.17), oral (HR = 2.80), and small intestine (HR = 3.90), all *p*s < 0.001.

**FIGURE 1:**
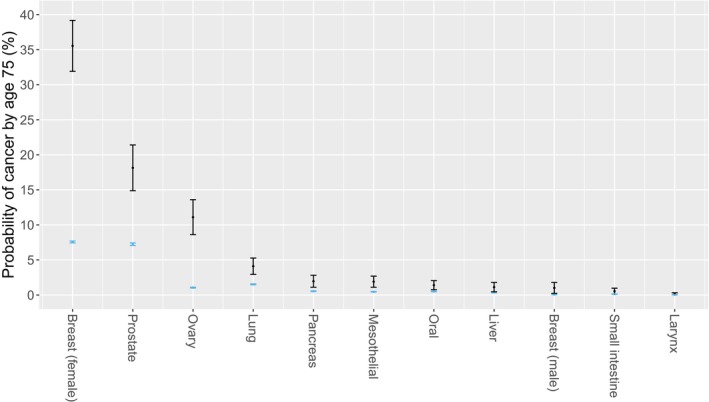
Predicted probability of cancer by age 75 years (P_75_) for *BRCA2*‐associated cancers estimated from *BRCA2*‐only models in the UK Biobank among carriers (black) and noncarriers (blue) of *BRCA2* PVs. Dots and whiskers represent point estimate and 95% confidence interval of prevalence, respectively.

The P_75_ was consistently higher in *BRCA2* PV carriers than noncarriers for each type of these 10 cancers (Figure 1). Among carriers, the P_75_ was substantially high for several core HBOC cancers, including female breast cancer (35.54%), prostate cancer (18.14%), and ovarian cancer (11.10%). Notably, the P_75_ in carriers for lung cancer (4.10%), a non‐HBOC cancer, was higher than that of two core HBOC cancers: pancreatic cancer (1.96%) and male breast cancer (1.01%).

### 3.4 Interplay of BRCA2 PVs and PGS on Cancer Risk

For *BRCA2*‐associated cancers where validated cancer‐specific PGS are available (breast, prostate, pancreas, ovary, larynx, lung, and oral), each PGS was associated with the respective cancer risk, independent of *BRCA2* PV carrier status, *p* < 0.001 (Table 2). Furthermore, a significant interaction effect between *BRCA2* PV status and PGS on cancer risk was observed for two cancers: female breast cancer (*p*_interaction_ < 0.001) and prostate cancer (*p*_interaction_ = 0.04). The association between PGS and the risk for each of these two cancers differed significantly between carriers and noncarriers where the strength (coefficient) of association of PGS with cancer was weaker (lower) among carriers than noncarriers in both cancers (Table S5), *p* < 0.05.

**TABLE 2:** Interplay of *BRCA2* PVs status and PGS on cancer risk.

	HR (95% CI) ^a^, ^b^	*p* value ^a^, ^b^
Breast (female), C50 ^c^		
*BRCA2*	5.49 (4.82–6.25)	3.25E‐145
PGS	1.81 (1.78–1.83)	< 1E‐300
*BRCA2* × PGS	0.79 (0.69–0.89)	2.03E‐04
Prostate, C61		
*BRCA2*	2.68 (2.20–3.27)	1.52E‐22
PGS	1.98 (1.95–2.02)	< 1E‐300
*BRCA2* × PGS	0.83 (0.70–0.99)	0.04
Breast (male), C50 ^d^		
*BRCA2*	13.99 (5.90–33.16)	2.13E‐09
PGS	1.34 (1.13–1.59)	8.46E‐04
*BRCA2* × PGS	1.18 (0.57–2.43)	0.65
Ovary, C56		
*BRCA2*	11.05 (8.65–14.11)	1.38E‐82
PGS	1.25 (1.20–1.31)	1.22E‐24
*BRCA2* × PGS	1.04 (0.82–1.32)	0.72
Pancreas, C25		
*BRCA2*	3.75 (2.40–5.86)	7.24E‐09
PGS	1.40 (1.34–1.47)	7.08E‐44
*BRCA2* × PGS	0.91 (0.59–1.39)	0.65
Oral, C00‐C14		
*BRCA2*	2.57 (1.52–4.34)	4.25E‐04
PGS	1.42 (1.36–1.48)	4.22E‐60
*BRCA2* × PGS	1.16 (0.78–1.71)	0.46
Larynx, C32, C33		
*BRCA2*	5.48 (2.35–12.79)	8.47E‐05
PGS	2.27 (2.11–2.45)	5.42E‐105
*BRCA2* × PGS	0.59 (0.28–1.22)	0.16
Lung and bronchus, C34		
*BRCA2*	2.76 (2.05–3.72)	2.43E‐11
PGS	1.40 (1.36–1.44)	1.85E‐131
*BRCA2* × PGS	0.96 (0.71–1.28)	0.77

^a^ Adjusted for gender and genetic background (top 10 PCs).

^b^ Time from birth date to event/death/the end of follow‐up (June 12, 2023).

^c^ Tested in female subjects.

^d^ Tested in male subjects.

**FIGURE 2:**
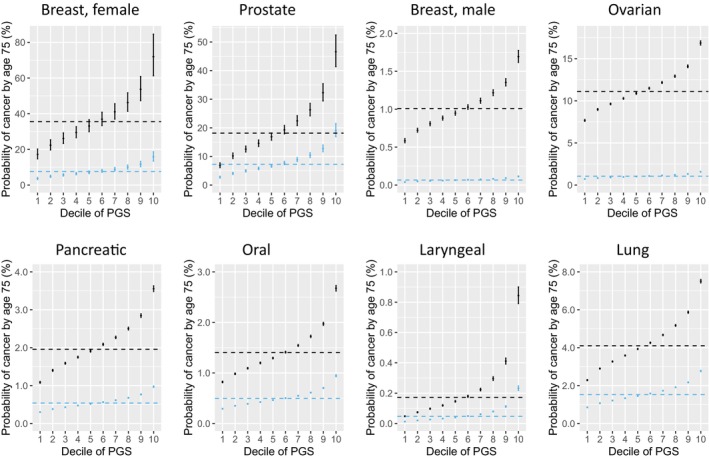
Predicted probability of cancer by age 75 years (P_75_) for *BRCA2*‐associated cancers estimated from *BRCA2* + PGS models in the UK Biobank among carriers (black) and noncarriers (blue) of *BRCA2* PVs by PGS decile. Dots and whiskers represent point estimate and 95% confidence interval of prevalence, respectively. Horizontal black and blue dotted lines indicate average P_75_ for carriers and noncarriers, respectively.

Correspondingly, the P_75_ for each of these seven cancers increased with higher cancer‐specific PGS deciles in both carriers and noncarriers (Figure 2). For several cancers, including female breast, prostate, lung, oral, and larynx, the difference in P_75_ between high‐ and low‐PGS deciles was as large as that between carriers and noncarriers. As a result, the P_75_ in carriers with lower PGS deciles was lower than that of noncarriers with higher PGS deciles. For example, the P_75_ for prostate cancer in carriers with the lower five PGS deciles ranged from 6.87% to 16.88%, which was lower than that of noncarriers with the highest PGS decile (19.06%).

### 3.5 Accuracy of Predicted Probability by *BRCA2* and PGS

**FIGURE 3:**
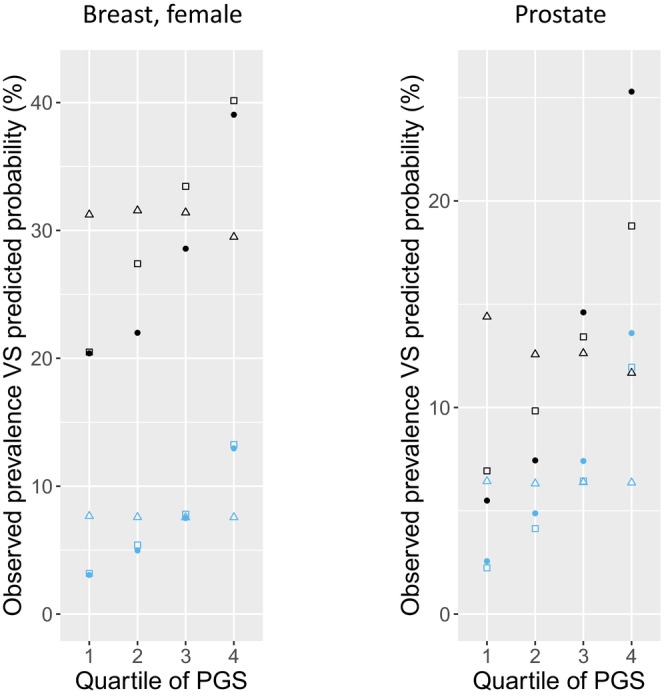
Comparison of observed cancer prevalence with predicted probability for female breast cancer and prostate cancer in the testing subset of the UKB (50% of UKB). Results for observed cancer prevalence (solid round dot) and predicted probability from two models (*BRCA2*‐only, open triangle, and *BRCA2* + PGS, open square, derived from the development subset of 50% subjects randomly selected from the UKB) are plotted for carriers (black) and noncarriers (blue) by PGS quartile.

To assess the accuracy of cancer risk assessment using *BRCA2* and cancer‐specific PGS, we developed two Cox regression models (*BRCA2*‐only and *BRCA2* + PGS, both include age, gender, and top 4 PCs) for the two most common HBOC cancers (female breast cancer and prostate cancer) in a randomly selected 50% of the subjects. We then estimated the probability of each cancer from these two models for the remaining 50% of the subjects and compared it to the observed cancer prevalence (Figure 3). The need for supplementing PGS to *BRCA2* PV status for more accurate risk assessment is evident by comparing the predicted probability from *BRCA2*‐only (open triangles) and *BRCA2* + PGS (squares) with observed cancer prevalence (solid dots). First, the observed prevalence was higher in carriers than noncarriers but markedly increased with higher PGS quartiles in both groups. Second, the probability estimated from the *BRCA2*‐only model was, however, flat across the PGS quartiles and deviated greatly from the observed prevalence, particularly in the lowest and highest quartiles. Third, the probability from the *BRCA2* + PGS model, on the other hand, aligned more closely with the observed prevalence across PGS quartiles in both carriers and noncarriers.

## 4 Discussion

Utilizing a large population based cohort with extensive genetic data and information on the diagnosis of all common cancers, we obtained several pieces of evidence for *BRCA2*‐associated cancers. We first showed that the spectrum of *BRCA2*‐associated cancers is not limited to core HBOC cancers but includes six additional types of cancer. We also demonstrated that the penetrance of cancers in carriers of *BRCA2* PVs increased significantly with higher cancer‐specific PGS deciles. Furthermore, we found a significant interaction effect between *BRCA2* and PGS on the risk of two cancers (female breast and prostate) where the PGS effect was weaker in carriers than in noncarriers. Finally, we illustrated the need to incorporate PGS in estimating penetrance for all *BRCA2*‐associated cancers.

Association of *BRCA2* PVs with cancers beyond core HBOC syndromes remains controversial [13]. This is likely due to a combination of factors such as relatively low penetrance for these cancers, rarity of *BRCA2* PVs, and small sample size. Several published studies reported a positive association between *BRCA2* and non‐HBOC cancers, including melanoma [5, 8], stomach [7, 8, 10], esophagus [8], uterine [4, 9], endometrial [11, 12], and leukemia [6]. For some of these cancers, increased risk was observed in our study, but failed to reach statistical significance after the Bonferroni correction for 53 tests (*p* < 1E‐04), including stomach (HR = 2.10, *p* = 0.03), esophagus (HR = 2.07, *p* = 0.02), and uterine cancer (HR = 1.69, *p* = 0.05). For melanoma, no evidence was found in our study (HR = 1.04, *p* = 0.88) despite a large number of melanoma cases (*N* = 5326). Similarly, no significant association was found for various types of leukemia. Among the six novel *BRCA2*‐associated cancers found in our study, the statistical evidence was notably strong for lung cancer and mesothelioma (*p* < 1E‐10), but moderate for oral, liver, small intestine, and laryngeal cancers. However, false positives are possible for these cancers, given the limited number of prior reports and the relatively small sample sizes (< 50 patients for each of these cancers among carriers). Additional large‐scale studies are needed to validate these associations.

An interesting and practically important observation of our study was the relatively lower penetrance for core HBOC cancers among carriers of *BRCA2* PVs than commonly estimated. For example, the P_75_ for female breast cancer was 35.54% (95% CI: 31.91%–39.18%), lower than that previously reported from two meta‐analyses where the cumulative risks of breast cancer by age 70 years was 55%–65% [17, 42]. A major factor for the difference is differing study designs. Penetrance in previous studies was typically estimated from highly selected subjects based on a strong family history of cancer, early age at cancer diagnosis, and multiple primary cancers [19]. In comparison, our study is a population based cohort where *BRCA2* carriers were systematically identified in all participants, where the vast majority of carriers did not report a family history of cancer. As such, higher estimated penetrance from previous studies is more appropriate for carriers who met the National Comprehensive Cancer Network (NCCN) guidelines for genetic testing of HBOC genes [3], while our estimates likely better reflect risk for unselected carriers.

A prominent finding of our study is that cancer penetrance in carriers of *BRCA2* PVs is significantly modified by cancer‐specific PGS. This is expected considering that PGS is an informative tool for cancer risk stratification and is generally independent of monogenic genes [22, 23, 24, 25, 26, 27]. It is also consistent with findings from previous studies in the general population [28, 29, 30, 31, 32, 33, 34, 35, 36, 37]. The substantial difference in penetrance among carriers with high‐ versus low PGS in our study strongly suggests that PGS should be included in penetrance estimations for carriers. This is particularly important for cancers such as female breast, prostate, and lung, where the difference between high‐ and low PGS exceeds the difference between carriers and noncarriers.

A notable observation is a weaker effect of PGS on cancer risk in carriers compared to noncarriers, suggesting an interaction effect between them. This observation was not found in the study of Fahed et al., where fewer *BRCA2* carriers (*N* = 845) and breast cancer patients (*N* = 1920) were analyzed [31]. However, a similar observation was reported for breast cancer by Gallagher et al. [32], and for colorectal cancer by Jenkins et al. [43] In the latter study, the well‐established colorectal cancer PGS was not significantly associated with colorectal cancer risk among the 826 carriers (HR = 0.97, *p* = 0.51). This finding has important clinical implications for genetic counseling in *BRCA2* carriers, as their risk may be overestimated if the carrier‐specific effect of PGS is not considered.

Our findings have several potential clinical implications for medical geneticists, primary care physicians, as well as patients. For carriers of *BRCA2* PVs, cancer risk assessment should extend beyond the core HBOC cancers to include six additional cancer types. The risk for *BRCA2*‐associated cancers should be evaluated in combination with cancer‐specific PGS. Furthermore, for female breast cancer and prostate cancer, a more accurate estimation of penetrance should use the carrier‐specific effect of PGS, rather than relying on the effect estimated from the general population. Although additional studies, particularly in minority populations, would be beneficial, the available data support the use of PGS in clinical settings for more accurate cancer risk assessment among carriers. This proposition is grounded in the principles of comparative effectiveness research (CER), which emphasize comparing the performance of new tools (*BRCA2* + PGS model) with existing ones (*BRCA2*‐only model), rather than aiming for perfection [44, 45]. Nevertheless, practical implementations of combining PGS with monogenic testing remain challenging despite the availability of clinical‐grade tests using combined panel sequencing, SNP arrays, and blended genome and exome (cBGE) assays. Key barriers include limited physician preparedness to interpret PGS and integrated genetic test results, as well as the absence of clear recommendations in current clinical guidelines. Initial efforts could focus on integrating existing risk assessment tools—such as CanRisk, which incorporates PGS into the BOADICEA model—to facilitate clinical adoption and improve interpretation of comprehensive genetic risk [46].

Several important limitations of the study are noted. First, despite the large sample size of the UKB, the number of carriers of *BRCA2* PVs remains limited, reducing the statistical power to detect associations for some rare cancer types. Therefore, caution is needed when interpreting null associations. Second, most of the results from this study are likely driven by subjects of European ancestry, who represent 94% of the UKB sample. However, these findings may have the potential to be generalized to other ancestries. For instance, the main effect of *BRCA2* (HR 4.18) and PGS (HR 1.63) on female breast cancer in non‐European subjects was also statistically significant, *p* < 0.001 (Table S6). Third, some cancer‐specific PGS may be prone to overfitting due to the partial contribution of UKB subjects to both GWAS and the PGS model development/validation. However, the impact of this potential bias on the results is likely minimal, as these PGS models have been independently validated in other cohorts, such as the Genomic Health Initiative (GHI), a hospital‐based biobank at Endeavor Health (Table S2). Fourth, the validation of our two prediction models may be prone to overfitting because the 50% testing cohort was drawn from the same population as the development cohort. To improve generalizability, validation using out‐of‐sample cohorts is necessary. Fifth, we did not include family history in the study because it was limited in the UKB. Family history was based on a self‐reported questionnaire about cancers in parents and siblings and was only available for breast, prostate, and bowel cancers. Finally, we acknowledge the limited scope of this *BRCA2*‐focused study. This approach was chosen due to the substantial differences in cancer risk profiles between *BRCA2* and *BRCA1*, as well as the greater number of *BRCA2* P/LP carriers in the UK Biobank, which allowed for increased statistical power. Similar analyses for *BRCA1* and other HBOC‐associated genes are warranted.

In conclusion, utilizing this large population based cohort, we implicated the risk of *BRCA2* PVs for classic HBOC cancers and six other types of cancer and demonstrated the interplay between *BRCA2* and PGS for cancer risk. These findings provide a strong rationale for incorporating PGS in cancer risk assessment among carriers of *BRCA2* PVs, highlighting its utility in refining penetrance estimates and improving clinical decision making.

## Author Contributions

Concept and design: **Jianfeng Xu**. Data analysis: **Zhuqing Shi**, **Huy Tran**, **Jun Wei**, **Chi‐Hsiung Wang**. Manuscript draft: **Brendan Prassas** and **Jianfeng Xu**. Critical revision of the manuscript for important intellectual content: all coauthors. Supervision: **Jianfeng Xu**.

## Ethics Statement

This study was based on the UK Biobank (access number: 50295). Ethical approval of UK Biobank is granted by the North West Multi‐centre Research Ethics Committee (MREC) as a Research Tissue Bank (RTB). Researchers using UK Biobank data do not require separate ethical clearance for their research.

## Consent

The UK Biobank oversees the rigorous consenting of all subjects and is responsible for following ethical guidelines.

## Conflicts of Interest

NorthShore University HealthSystem has agreements with GenomicMD and GoPath Laboratories for genetic tests of polygenic risk scores. J. Xu serves as a scientific advisory board member for GoPath Laboratories and GenomicMD.

## Supporting information


**Data S1.** Supporting Information.

## Data Availability

The data that support the findings of this study are available from the corresponding author upon reasonable request.
